# Breast cancer: the first comparative evaluation of oncobiome composition between males and females

**DOI:** 10.1186/s13293-023-00523-w

**Published:** 2023-06-05

**Authors:** Elena Niccolai, Simone Baldi, Giulia Nannini, Francesca Gensini, Laura Papi, Vania Vezzosi, Simonetta Bianchi, Lorenzo Orzalesi, Matteo Ramazzotti, Amedeo Amedei

**Affiliations:** 1 Department of Experimental and Clinical Medicine, University of Florence, Largo Brambilla 3, 50134 Florence, Italy; 2 Department of Biomedical, Experimental and Clinical Sciences “Mario Serio”, University of Florence, Florence, Italy; 3 Division of Pathological Anatomy, Department of Health Sciences, University of Florence, Florence, Italy

**Keywords:** Oncobiome, FFPE, Dimorphism, Microbiota, Breast cancer, Gender

## Abstract

**Background:**

Emerging evidence suggests that breast microbiota dysbiosis contributes to cancer initiation, progression, prognosis and treatment efficacy. Anyway, available data are referred only to female patients, and studies on males are completely missing. Male breast cancer (MBC) is 70–100 times less frequent, but the mortality rate adjusted to incidence is higher in men than in females. Currently, MBC diagnostic approaches and treatments have generally been extrapolated from the clinical experience gained in women, while few studies focus on characterizing male cancer biology. Taking into account the rising importance of the oncobiome field and the need of MBC targeted studies, we explored the breast cancer oncobiome of male and female patients.

**Methods:**

16S rRNA gene sequencing was performed in 20 tumor and 20 non-pathological adjacent FFPE breast tissues from male and female patients.

**Results:**

We documented, for the first time, the presence of a sexually dimorphic breast-associated microbiota, here defined as “breast microgenderome”. Moreover, the paired analysis of tumor and non-pathological adjacent tissues suggests the presence of a cancer-associated dysbiosis in male patients, with surrounding tissue conserving a healthier microbiome, whereas in female patients, the entire breast tissue is predisposed to cancer development. Finally, the phylum Tenericutes, especially the genera *Mesoplasma* and *Mycobacterium*, could to be involved in breast carcinogenesis, in both sexes, deserving further investigation, not only for its role in cancer development but even as potential prognostic biomarker.

**Conclusions:**

Breast microbiota characterization can enhance the understanding of male breast cancer pathogenesis, being useful for detection of new prognostic biomarkers and development of innovative personalized therapies, remarking the relevant gender differences.

**Supplementary Information:**

The online version contains supplementary material available at 10.1186/s13293-023-00523-w.

## Plain Language Summary

Breast tissue can become inhabited by microbes through different pathways, and an uneven distribution of these microorganisms could potentially contribute to the development, prognosis, and treatment response of breast cancer. However, the current available data primarily focus on female patients, with a significant dearth of studies on males. To address this gap, the present study investigates the microbiota composition of both tumorous and healthy breast tissue samples from both male and female patients.

The findings of this research highlight a disparity in the types of bacteria present in male and female breast tissue. Specifically, it shows that male patients with breast cancer have a higher imbalance of bacteria in the cancerous area compared to the surrounding healthy tissue. In contrast, in females the dysbiosis extend to the whole breast tissue.

Moreover, the study identifies specific strains of bacteria that might potentially be involved in the development of breast cancer in both males and females.

In conclusion, this study underscores the significance of microbial colonization in breast tissue and its potential influence on breast cancer in both males and females. By expanding our understanding of the microbial composition in breast cancer, we can pave the way for innovative diagnostic methods and treatment approaches for male breast cancer, while simultaneously advancing our knowledge of this complex disease.

**Supplementary Information:**

The online version contains supplementary material available at 10.1186/s13293-023-00523-w.

## Highlights

1. Few studies have been conducted to describe male cancer biology in comparison with female breast cancer.
2. For the first time, we discovered a sexually dimorphic breast-associated microbiome, termed “breast microgenderome”.
3. In females, the dysbiosis extend to the whole breast tissue, while in men, it appears to be confined to the tumor site.
4. Tenericutes, particularly the genera *Mesoplasma* and *Mycobacterium*, may be implicated in breast carcinogenesis in both sexes.

**Supplementary Information:**

The online version contains supplementary material available at 10.1186/s13293-023-00523-w.

## Introduction

Incidence and mortality for breast cancer are quite similar among developed countries, but vary widely across genders. Female breast cancer (FBC) is actually the most common metastatic malignancy and the first cause of cancer death for women worldwide [1]. The most common FBC risk factors include aging, family history, lifestyle and exposure to endogenous or exogenous estrogens [2]. Based on gene expression profiling, FBC are classified in different subtypes useful for predicting prognosis and guiding the proper clinical treatment (antiestrogen therapy, targeted therapy, chemotherapy, or combined immunotherapeutic approaches) [3, 4]. Contrariwise, male BC (MBC) is a rare and still incompletely defined disease usually characterized by BRCA2 rather than BRCA1 mutations [5]. Less than 1% of all breast cancer cases occur in men and the main risk factors are represented by Klinefelter syndrome, obesity, alcohol abuse and occupational hazards [6]. Male breast tissue generally do not differentiate and do not present lobule formation and the predominant histological type of MBC is invasive no special type (NST) accounting for more than 90% of all MBC [7]. Regarding the prognosis, no sex differences were found between age-matched and stage-matched BCs and less favorable outcome in men are determined by more advanced stage of tumor presentation and the probable delay in diagnosis due to the low suspicion index of both patients and clinicians [8, 9]. MBC is 70–100 times less frequent than FBC, but the mortality rate adjusted to incidence is higher in men than in females [10]. Moreover, while mortality rates in women are decreasing, the same does not hold true for men, suggesting that current treatments for MBC are less efficient [11].

The discovery of a resident breast microbiota that varies among non-pathological breast and cancer, paired adjacent non-pathological and tumoral tissues, as well as benign and malignant breast diseases, suggests the oncobiome role in breast carcinogenesis and its potential role in predicting the cancer risk [12, 13]. Specifically, the established link between the intratumoral microbiota and the immune cell infiltrate composition, indicates that dysbiosis should contribute to tumor immune evasion [14] and to breast cancer treatment resistance [15, 16]. Actually, distinct oncobiomes have been associated with different FBC subtypes, providing diagnostic and prognostic information on treatment efficacy that can be critical for personalize therapeutic interventions [17-19]. But, no similar studies are available for MBC patients, especially because of the disease rarity.

In the present study, exploiting available histopathological Formalin fixed paraffin-embedded (FFPE) samples, we characterized and compared, for the first time, the microbiome composition of non-pathological adjacent and tumor breast tissues of male and female patients, in order to reveal the existence of a sexual dimorphism that may underlie differences in cancer progression and sensitivity to treatments.

## Materials and methods

### Samples collection

40 FFPE breast tissue samples were obtained from 20 patients (10 males and 10 females) diagnosed with BC (Table 1) at “Careggi University Hospital” (Florence, Italy) from September 2011 to February 2020. Males had a median age of 72 (IQR 55–82) and females of 47 (IQR 41–66), with a substantial disparity between the two groups (*p* = 0.011, *t* test).

**Table 1:** Patients’ clinical and demographical characteristics

Patient ID	Age	Gender	Diagnosis	Tumor differentiation	Regional lymph nodes	Estrogen receptor (SP1)	Progesterone receptor (1E2)	Ki67	c-erb-B2
BC1	70	F	Invasive lobular carcinoma	G2	pN0	100%	10%	15%	Score 1+
BC 2	64	F	Invasive lobular carcinoma	G2	pN3a	100%	10%	15%	Score 1+
BC 3	33	F	Invasive NSTNST carcinoma	G3	pN1a	100%	40%	60%	Score 0
BC 4	47	F	Invasive lobular carcinoma	G2	pN0	100%	90%	20%	Score 2+
BC 5	42	F	Invasive NST carcinoma	G3	pN0	100%	80%	80%	Score 1+
BC 6	46	F	Invasive NST carcinoma	G2	pN0	100%	100%	25%	Score 2+
BC 7	48	F	Invasive papillary carcinoma	G3	pN0	100%	90%	25%	Score 2+
BC 8	42	F	Invasive NST carcinoma	G2	pN1a	100%	100%	30%	Score 2+
BC 9	39	F	Invasive NST carcinoma	G3	pN0	Negative	Negative	80%	Score 0
BC 10	79	F	Invasive lobular carcinoma	G2	pN0	100%	90%	15%	Score 2+
BC11	77	M	Invasive papillary carcinoma	G2	pN0	100%	90%	25%	Score 2+
BC 12	48	M	Invasive NST carcinoma	G2	pNx	100%	100%	20%	Score 0
BC 13	57	M	Invasive NST carcinoma	G3	rpN0	100%	100%	40%	Score 2+
BC 14	48	M	Invasive NST carcinoma	G3	rpN0	90%	80%	70%	Score 2+
BC 15	69	M	Invasive NST carcinoma	G3	pN0	90%	30%	40%	Score 2+
BC 16	68	M	Invasive NST carcinoma	G3	pN2a	100%	100%	30%	Score 0
BC 17	76	M	Invasive NST carcinoma	G2	pN0	90%	10%	15%	Score 0
BC 18	84	M	Invasive NST carcinoma	G3	pN2a	100%	90%	40%	Score 2+
BC 19	88	M	Invasive NST carcinoma	G2	pNx	100%	100%	25%	Score 2+
BC 20	82	M	Invasive NST carcinoma	G2	pN1a	90%	60%	25%	Score 1+

For each sample, the first few scrolls of the FFPE blocks were discarded to minimize the environmental contamination, and then eight/ten sections, each with a thickness of up to 10 µm and a surface area of up to 250 mm^2^, obtained through microtome, where collected into sterile 2-ml centrifuge tubes. To control for potential contamination in downstream analysis, paired empty paraffin from the same FFPE tissue block was collected. The microtome was cleaned between patient samples and the equipment was regularly tested using sterile swabs.

### Characterization of breast tissues microbiota

Genomic DNA was extracted using the QIAmp DNA FFPE Advanced Kit (Qiagen, Hilden, Germany) from male tumor (MT) and non-pathological adjacent (MH) tissues, from female tumor (FT) and adjacent non-pathological (FH) tissues, and from empty paraffin, according to the manufacturer’s instructions.

The quality and quantity of extracted DNA was assessed using the NanoDrop ND-1000 (Thermo Fisher Scientific, Waltham, US) and the Qubit Fluorometer (Thermo Fisher Scientific). Extracted DNA samples were sent to IGA Technology Services (Udine, Italy) where amplicons of the variable V3–V4 region of the bacterial 16s rRNA gene were sequenced in paired-end (2 × 300 cycles) on the Illumina MiSeq platform, according to the Illumina 16S Metagenomic Sequencing Library Preparation protocol. Lastly, the raw data were processed following the software pipeline MICCA as we previously described [20].

Statistical analyses on the bacterial communities were performed in R [21] with the help of the packages phyloseq 1.26.1 [22], decontam 1.2.1, DESeq2 1.22.2 [23], and other packages satisfying their dependencies, in particular, vegan 2.5-5 [24]. An initial screening step was performed using Bowtie2 [25] to identify and remove unwanted and non-specific human amplicons (due to the biomass unbalance between human and microbial components). For further identifying possible contaminants (e.g., due to tissue manipulation or paraffin) the decontam package was used in the “combined” mode (that required both the concentration of the DNA in original extracts and at least one negative control) and setting the stringency filter to 0.5. For the cluster analysis of the entire community, the abundance tables at the different ranks was first normalized using the total counts of each sample and then adjusted using square root transformation. Shannon, Chao 1 and evenness indices were used to estimate bacterial diversity in each sample using the function estimate_richness from phyloseq. The evenness index was calculated using the formula *E* = S/log(*R*), where S is the Shannon diversity index and *R* is the number of OTUs in the sample. Differences in all indices between grouped samples were tested using a paired and not paired Wilcoxon tests and *p*-values less than 0.05 were considered statistically significant. The permutational ANOVA (PERMANOVA) test was applied to beta-diversity distance matrices to test significance between samples’ clusters observed following principal coordinate analysis (PCoA); significance was determined through 999 permutations.

The differential abundance analysis at the different taxonomic ranks (created using the tax_glom function in phyloseq) was performed with DESeq2 [23] using a two-group blocked by patient design in order to perform a paired test when needed. Fold changes in differential analysis was calculated using the shrinked form using the ‘apeglm’ method [26].

## Results

### Preliminary data: preprocessing and cleaning process

In order to compensate for possible microbial contaminants that have been previously documented in paraffin samples [27], we screened the OTU abundance table, originally composed by 1912 OTUs, with the R package decontam (using the frequency mode and a threshold of 0.5), that indicated 301 OTUs as possible contaminants. Additionally, since the biomass of microbes was low with respect to human tissues, we screened the OTU sequences for human contamination using Bowtie2, resulting in 184 spurious OTUs. After removal of human sequences and contaminants, a final collection of 1437 microbial OTUs was obtained. Overall, 26 phyla, 51 classes, 87 orders, 185 families and 445 genera were identified across all samples.

### Breast tissues-associated microbiota differs in male and female patients

**Fig. 1:**
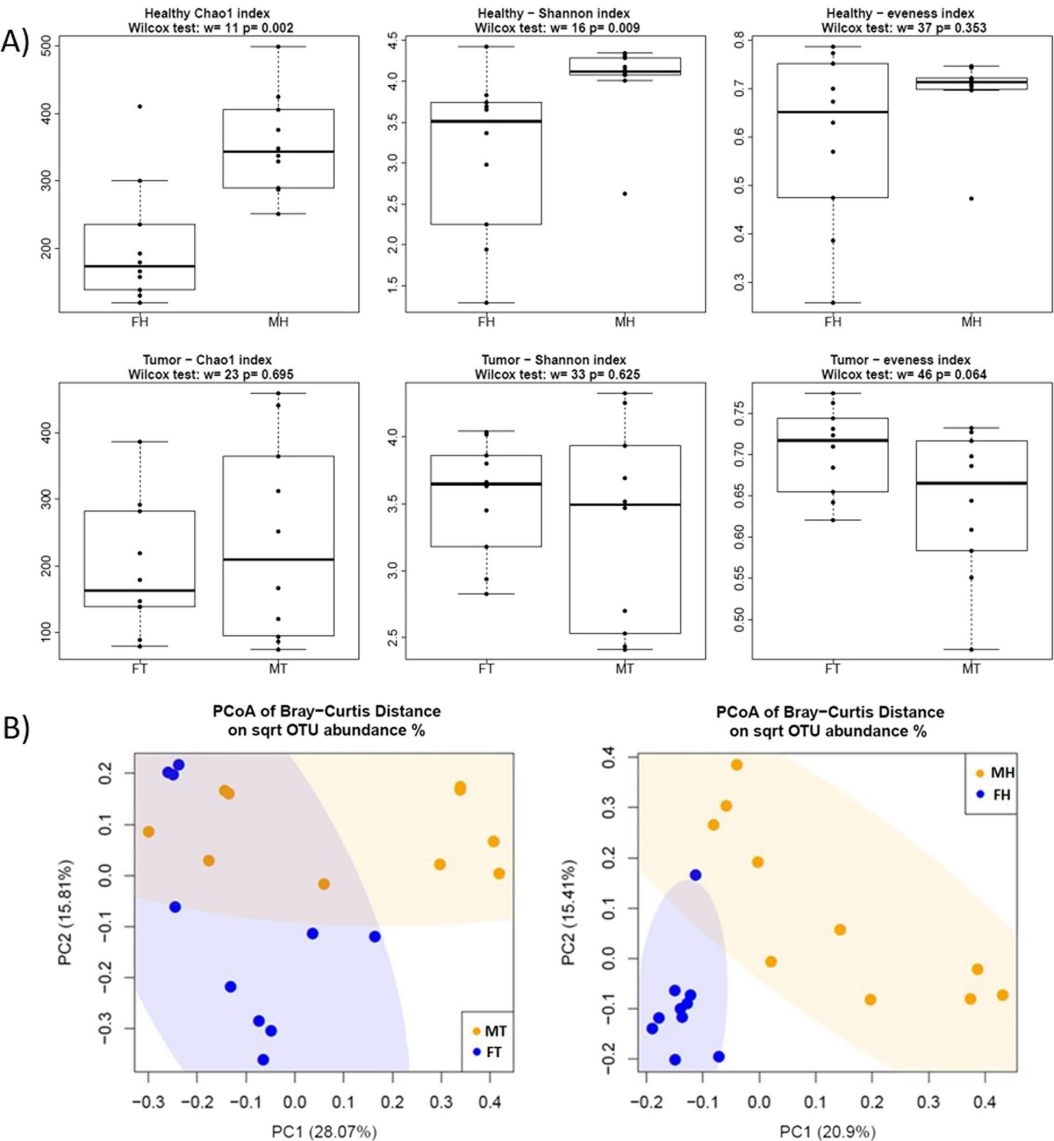
Alpha and beta-diversity analysis between males and females breast tissues. **A** Boxplots showcasing alpha diversity indices (Chao1 index, Shannon index, evenness). **B** Principal coordinates analysis (PCoA) according to the Bray–Curtis beta-diversity metric of male and female breast tissues. *FH* female adjacent non-pathological samples, *FT* female tumor samples, *MH* male adjacent non-pathological samples, *MT* male tumor samples

**Fig. 2:**
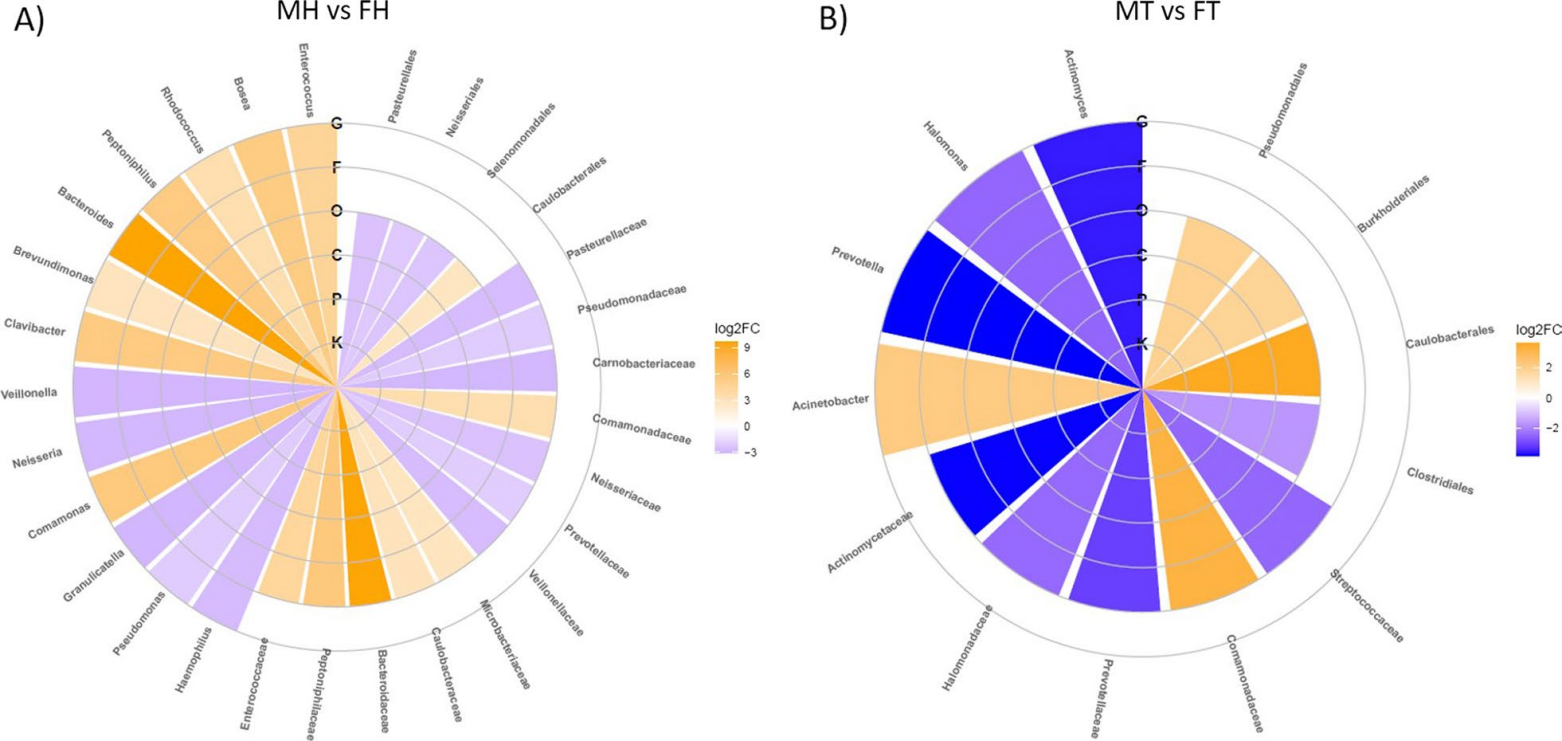
Circular heatmaps of differentially abundant taxa. **A** The differentially abundant taxa between MH and FH samples; **B** the differentially abundant taxa between MT and FT samples. Concentric circles represent taxonomic ranks. Yellow shades indicate positive logFC values, whereas blue shades indicate negative logFC values correlations; the intensity of colors is proportional to logFC values. *FH* female adjacent non-pathological samples, *FT* female tumor samples, *MH* male adjacent non-pathological samples, *MT* male tumor samples. *K* kingdom, *P* phylum, *C* class, *O* order, *F* family, *G* genus, *FC* fold change

Male and female patients displayed a different breast’s microbiota composition, both in non-pathological and tumor tissues. Comparing the non-pathological breast tissues, through the analysis of alpha diversity (the measure of microbiome diversity within a sample), male tissues exhibited a significantly greater microbial diversity and richness (Chao 1, *p* = 0.002; Shannon, *p* = 0.009) compared to female ones (Fig. 1A). In addition, the beta-diversity (estimation of the similarity of two communities) through PCoA using Bray–Curtis distance showed that male non-pathological samples clustered away from females (PERMANOVA, Pr(> *F*) = 0.001, Fig. 1B). Accordingly, the DESeq2 analysis showed that, 4 orders, 12 families and 13 genera were differentially abundant (Fig. 2A, Additional file 1: Table S1). Regarding tumor samples, differences among sexes were found to a lesser extent. Alfa indices were not significantly different, but tumor male and female samples clustered separately on PCoA plot according to beta-diversity (PERMANOVA, Pr(> *F*) = 0.01) (Fig. 1A, B). Regarding the taxonomic composition of tumor samples, no difference was observed at phylum levels, where the two most abundant phyla were Proteobacteria and Firmicutes in both cases. Besides, at lower taxonomic levels we found that 4 orders, 5 families and 4 genera displayed unequal frequencies in male vs female patients (Fig. 2B, Additional file 1: Table S2).

### Tumor and paired non-pathological breast tissues’ microbiota diverges significantly only in male cancer patients

**Fig. 3:**
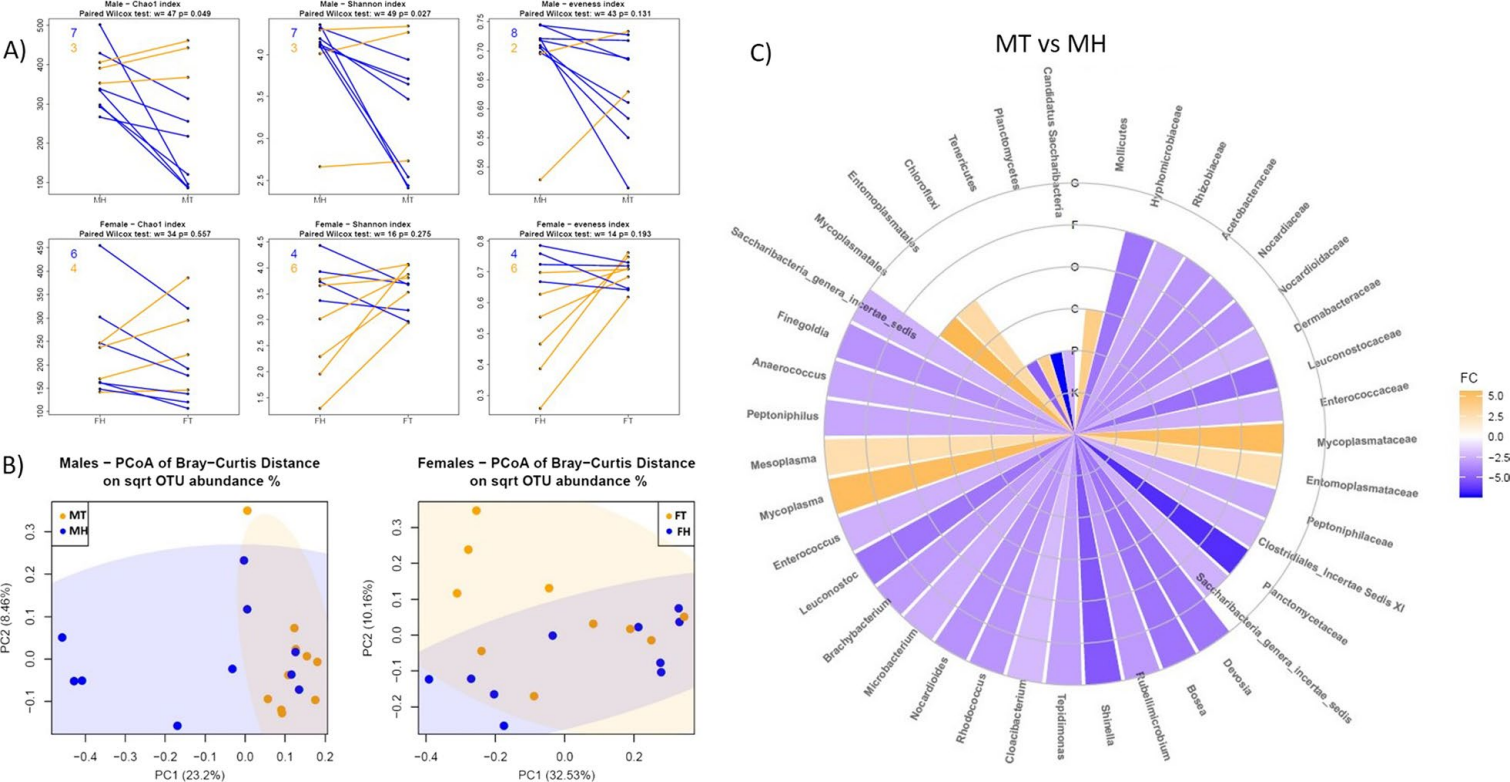
Breast microbiota in male and female tumor tissues compared with matched non-pathological adjacent tissues. **A** Segment plots displaying alpha diversity indices (Chao1 index, Shannon index and evenness). The numbers in the top left corner represent counts of decreased (blue) and increased (yellow) measurements for paired samples. **B** Principal coordinates analysis (PCoA) according to the Bray–Curtis beta-diversity metric of male and female breast tissues. **C** Circular heatmap representing the differentially abundant taxa in male tumor versus non-pathological adjacent tissues: concentric circles represent taxonomic ranks from kingdom to genus; yellow shades indicate positive logFC values, whereas blue shades indicate negative logFC values correlations; the intensity of colors is proportional to logFC values. *FH* female adjacent non-pathological samples, *FT* female tumor samples, *MH* male adjacent non-pathological samples, *MT* male tumor samples. *K* kingdom, *P* phylum, *C* class, *O* order, *F* family, *G* genus, *FC* fold change

In male patients, tumor (MT) and paired non-pathological adjacent (MH) tissues displayed a different microbial community structure. Firstly, the analysis of alpha diversity indicated a reduced richness (Chao1, *p* = 0.049) and diversity (Shannon, *p* = 0.027) in MT compared to MH (Fig. 3A). Secondly, the principal coordinate analysis according to Bray–Curtis beta-diversity metric showed that the overall bacterial taxa composition was diverse (PERMANOVA, Pr(> *F*) = 0.001; Fig. 3B). Moreover, matched pairs analysis at all taxonomic ranks revealed significant differences in the relative abundances of 18 genera, 14 families, 2 orders, 1 class, and 3 phyla between tumor and adjacent normal tissue (Fig. 3C, Additional file 1: Table S3).

On the other hand, in female, tumor and non-pathological adjacent paired tissues did not show a significant discrepancy both in the microbial community structure (PERMANOVA, Pr(> *F*) = 0.144; Fig. 3A, B) as well as in the bacterial taxa composition (data not shown).

## Discussion

Considering that mammary dysbiosis seems to have an impact in female breast cancer biology and that similar information are missing in males, we analyzed the breast-associated microbiota composition of male and female cancer patients. Because breast cancer in men is uncommon, we capitalized formalin-fixed paraffin-embedded tissue specimens for a retrospective microbiota characterization. FFPE samples represent a precious resource of patient-related samples, that, if correctly laboring, could increase the sample sizes and sites available for the oncobiota studies [28].

First, we compared the breast microbiota composition of male and female patients, taking into account the different age between groups. Our data demonstrate the presence of a “breast microgenderome” [29], even in the oncobiome structure. Indeed, many differences were registered in the non-pathological breast, and to a lesser extent in the tumor tissues. A sexual dimorphism of breast microbiota could be expected for many reasons, since the differences in breast anatomy, immune responses, hormonal status, and even cancer biology, can shape the microbial community [30-33]. Above all, female breasts are characterized by a more nutrient-rich fatty composition, a widespread vasculature and a diffuse location of lobules and ducts leading from the nipple that favor bacterial to thrive [32]. Moreover, skin and oral bacteria, during lactation, can use the nipple to gain access to the breast ducts and join the local microbial community [33]. Furthermore, sex steroids can modulate the microbiota composition directly, altering bacterial physiology [34], and indirectly, modulating inflammation and adaptive immune response, that, in turn, shape the microbiota composition [35-37].

Anyway, the non-pathological breast tissues of male patients displayed a more varied and diverse microbial community compared to females, with the enrichment of several microbial taxa such as the order Caulobacterales, Bacteroidaceae, Caulobacteraceae, Comamonadaceae, Enterococcaceae, Microbacteriaceae and Peptoniphilaceae families and *Bacteroides Brevundimonas Clavibacter Comamonas* and *Rhodococcus* genera. Contrariwise, the non-pathological female breast tissue showed higher levels of Pasteurellales, Neisseriales and Selenomonadales (genera *Haemophilus*, *Pseudomonas*, *Neisseria*, *Veillonella*). Regarding the cancer district, the male samples showed higher abundances of members of (i) orders Burkholderiales, Caulobacterales, and Pseudomonadales; (ii) families Comamonadaceae; and (iii) *Acinetobacter* spp. compared to females, and notably, many of these taxa (i.e., Comamonadaceae, Pseudomonadaceae, Caulobacteraceae members and *Acinetobacter radioresistens* were previously documented in human breast cancer tissue [12, 38]. On the other side, tumor female breast tissues showed higher levels of Clostridiales, Actinomycetaceae (*Actinomyces* spp.), Halomonadaceae (*Halomonas* spp.), Prevotellaceae (*Prevotella* spp.) and Streptococcaceae.

Furthermore, performing a comparison of paired tumor and non-pathological tissues, we highlighted a different situation in male and female patients. In female patients, tumor and non-pathological adjacent microbiota had a comparable composition, while, in male patients, tumor tissues showed a decreased microbial diversity and a different microbial composition compared to non-pathological adjacent ones. A reduced bacterial diversity and richness is often associated with malignancy, and frequently documented in cancer compared to paired non-pathological tissues [12, 20]. Anyway, in agreement with our data, Urbaniak et al., although demonstrating a dysbiosis in female breast cancer patients compared to non-pathological controls, did not report differences in bacterial communities between breast tumor and paired normal tissue [39]. These findings could suggest that in females, there is a predisposition to carcinogenesis of the entire breast tissue, while in males a cancer-associated dysbiosis is more evident. Of note, male tumor samples showed an increased level of the phylum Tenericutes, especially of *Mycoplasma* spp. that is linked with carcinogenesis in various tumors [40]. The increasing abundance of this phylum in tumor compared to non-pathological tissue and in female compared to male, strongly suggest its implication in breast cancer development, in both sexes.

Overall, our study presents some limitations, such as the low taxonomical resolution of 16S rRNA sequencing, the limited number of samples, and the use of FFPE instead of fresh frozen specimens. Anyway, even if the FFPE samples utilization presents technical pitfall and concerns (i.e., the low biomass in tumor environment, the DNA alterations during fixing and embedding process; the environmental contamination) with the adoption of precautions and specific protocols, it offers the advantage of capitalize the histopathological samples that are routinely collected [41], especially for rare diseases like the MBC. Furthermore, as the primary focus of our data is on the differential abundance in bacterial taxon across the analyzed groups, the existence of an environmental contamination, which cannot be totally ruled out, is of secondary concern in the present study.

Aware of those limits, our pioneer and explorative study shows an undoubted novelty, documenting, for the first time, the presence of a breast-associated microgenderome. Moreover, the analysis of tumor and non-pathological adjacent tissues suggests that, in female patients, the entire breast tissue is predisposed to cancer development, whereas, in male, the dysbiosis is confined (or more pronounced) in the tumor microenvironment. Finally, the phylum Tenericutes, especially the genera *Mesoplasma* and *Mycobacterium*, could to be involved in breast carcinogenesis, in both sexes, deserving further investigation, not only for its role in cancer development but even as potential prognostic biomarker. Considering the possibility to develop strategies to target breast microbiota in order to improve breast treatment, especially in male, our findings can pave the way for innovative personalized therapies which consider that gender differences may affect patient preferences, toxic effects from therapies, and finally survivorship priorities.

## Perspective and significance

Aware of the above-mentioned limits, our pioneer and explorative study shows an undoubted novelty, documenting, for the first time, the presence of a breast-associated microgenderome. The analysis of tumor and non-pathological adjacent tissues suggests that, in female patients, the entire breast tissue is predisposed to cancer development, whereas, in male, the dysbiosis is confined (or more pronounced) in the tumor microenvironment. Furthermore, the phylum Tenericutes, especially the genera *Mesoplasma* and *Mycobacterium*, could to be involved in breast carcinogenesis, in both sexes, deserving further investigation, not only for its role in cancer development but even as potential prognostic biomarker. Considering the possibility to develop strategies to target breast microbiota in order to improve breast treatment, especially in male, our findings can pave the way for innovative personalized therapies which consider that gender differences may affect patient preferences, toxic effects from therapies, and finally survivorship priorities.

## Supplementary Information

**Additional file 1: Table S1.** Significant differentially abundant phyla, classes, orders, families and genera between MH and FH samples. **Table S2.** Significant differentially abundant classes, orders, families, and genera between MT and FT samples. **Table S3.** Significant differentially abundant phyla, classes, orders, families and genera between MT and MH samples.

## Data Availability

The datasets generated in this study are publicly available in NCBI Gene Expression Omnibus (GEO) repository at https://www.ncbi.nlm.nih.gov/geo/query/acc.cgi?acc=GSE212891.

## Abbreviations

FBC: Female breast cancer
MBC: Male breast cancer
NST: Non-special type
FFPE: Formalin fixed paraffin-embedded
PCoA: Principal coordinate analysis
OUT: Operational taxonomic unit
