# Supplementary material for: Breast cancer: the first comparative evaluation of oncobiome composition between males and females
Source: Biol Sex Differ. 2023 Jun 5;14:37. doi: 10.1186/s13293-023-00523-w (PMC10243058; doi:10.1186/s13293-023-00523-w)
Supplement: Supplementary file 1 — Additional file 1: Table S1. Significant differentially abundant phyla, classes, orders, families and genera between MH and FH samples. Table S2. Significant differentially abundant classes, orders, families, and genera between MT and FT samples. Table S3. Significant differentially abundant phyla, classes, orders, families and genera between MT and MH samples. [file 13293_2023_523_MOESM1_ESM.docx]

**Table S1.** Significant differentially abundant phyla, classes, orders, families and genera between MH and FH samples. The table report the Log2FoldChange and adjusted p-values assessed using Mann-Whitney test.

| **Log2FoldChange** | **padj** |  |  | |  |
| --- | --- | --- | --- | --- | --- |
| 2,6150 | 0,0239 | Caulobacterales | | **Order** | |
| -2,2375 | 0,0056 | Neisseriales | |  |  |
| -2,5052 | 0,0056 | Pasteurellales | |  |  |
| -2,3516 | 0,0239 | Selenomonadales | |  |  |
| 9,5695 | 1,73e-05 | Bacteroidaceae | | **Family** | |
| -2,9398 | 0,0010 | Carnobacteriaceae | |  |  |
| 2,8850 | 0,0037 | Caulobacteraceae | |  |  |
| 3,4382 | 0,0106 | Comamonadaceae | |  |  |
| 4,3268 | 0,0194 | Enterococcaceae | |  |  |
| 2,7287 | 0,0039 | Microbacteriaceae | |  |  |
| 5,7012 | 1,73e-05 | Peptoniphilaceae | |  |  |
| -2,7600 | 0,0038 | Pasteurellaceae | |  |  |
| -2,0686 | 0,0180 | Prevotellaceae | |  |  |
| -2,1005 | 0,0137 | Pseudomonadaceae | |  |  |
| -2,50389 | 0,0037 | Neisseriaceae | |  |  |
| -2,7604 | 0,0118 | Veillonellaceae | |  |  |
| 9,6489 | 1,35e-05 | *Bacteroides* | | **Genus** | |
| 2,7831 | 0,0140 | *Brevundimonas* | |  |  |
| 5,4363 | 7,27e-06 | *Clavibacter* | |  |  |
| 5,3730 | 0,0136 | *Bosea* | |  |  |
| 5,6460 | 0,0013 | *Comamonas* | |  |  |
| -3,0131 | 0,0013 | *Granulicatella* | |  |  |
| -2,7716 | 0,0013 | *Haemophilus* | |  |  |
| -2,9802 | 0,0008 | *Neisseria* | |  |  |
| 5,4713 | 7,27e-06 | *Peptoniphilus* | |  |  |
| -2,0855 | 0,0054 | *Pseudomonas* | |  |  |
| 3,4351 | 0,0451 | *Rhodococcus* | |  |  |
| -3,0025 | 0,0090 | *Veillonella* | |  |  |

**Table S2.** Significant differentially abundant classes, orders, families, and genera between MT and FT samples. The table report the Log2FoldChange and adjusted p-values assessed using Mann-Whitney test.

| **Log2FoldChange** | **padj** |  |  |
| --- | --- | --- | --- |
| 1,7986 | 0,0441 | Burkholderiales | **Order** |
| 3,6036 | 0,0425 | Caulobacterales |  |
| -1,7178 | 0,0329 | Clostridiales |  |
| 1,8851 | 0,0329 | Pseudomonadales |  |
| -3,7948 | 0,0325 | Actinomycetaceae | **Family** |
| 3,1888 | 0,0398 | Comamonadaceae |  |
| -2,4482 | 0,0398 | Halomonadaceae |  |
| -3,1312 | 0,0397 | Prevotellaceae |  |
| -2,5057 | 0,0398 | Streptococcaceae |  |
| 2,1504 | 0,0429 | *Acinetobacter* | **Genus** |
| -3,6291 | 0,0429 | *Actinomyces* |  |
| -2,5098 | 0,0429 | *Halomonas* |  |
| -3,8056 | 0,0429 | *Prevotella* |  |

| **Log2FoldChange** | **padj** |  |  |
| --- | --- | --- | --- |
| -2,3570 | 0,0039 | Candidatus Saccharibacteria | **Phylum** |
| -5,0626 | 0,0040 | Chloroflexi |  |
| -7,3379 | 3,04e-05 | Planctomycetes |  |
| 3,8958 | 6,42e-18 | Tenericutes |  |
| 3,9254 | 2,09e-14 | Mollicutes | **Class** |
| 2,9614 | 2,16e-05 | Entomoplasmatales | **Order** |
| 5,6228 | 1,12e-13 | Mycoplasmatales |  |
| -3,3091 | 0,0207 | Acetobacteraceae | **Family** |
| -2,4089 | 0,0261 | Clostridiales_Incertae Sedis XI |  |
| -2,3540 | 0,0411 | Dermabacteraceae |  |
| -2,5153 | 0,0414 | Enterococcaceae |  |
| 2,6650 | 0,0004 | Entomoplasmataceae |  |
| -4,3430 | 0,0001 | Hyphomicrobiaceae |  |
| -4,5015 | 0,0001 | Leuconostocaceae |  |
| 5,3431 | 1,43e-10 | Mycoplasmataceae |  |
| -3,3027 | 0,0001 | Nocardiaceae |  |
| -3,4213 | 0,0081 | Nocardioidaceae |  |
| -2,7720 | 0,0182 | Peptoniphilaceae |  |
| -6,5289 | 0,0001 | Planctomycetaceae |  |
| -2,7183 | 0,0182 | Rhizobiaceae |  |
| -2,4619 | 0,0261 | Saccharibacteria_genera_incertae_sedis |  |
| -2,7754 | 0,0404 | *Anaerococcus* | **Genus** |
| -4,4139 | 0,0077 | *Bosea* |  |
| -3,1451 | 0,0189 | *Brachybacterium* |  |
| -2,1783 | 0,0386 | *Cloacibacterium* |  |
| -4,3535 | 0,0001 | *Devosia* |  |
| -2,4936 | 0,0446 | *Enterococcus* |  |
| -3,4114 | 0,0189 | *Finegoldia* |  |
| -4,3500 | 0,0005 | *Leuconostoc* |  |
| 2,6075 | 0,0018 | *Mesoplasma* |  |
| -2,5670 | 0,0077 | *Microbacterium* |  |
| 5,3100 | 8,96e-10 | *Mycoplasma* |  |
| -3,4431 | 0,0175 | *Nocardioides* |  |
| -2,7553 | 0,0189 | *Peptoniphilus* |  |
| -3,3414 | 0,0001 | *Rhodococcus* |  |
| -3,1981 | 0,0077 | *Rubellimicrobium* |  |
| -2,4326 | 0,0386 | *Saccharibacteria_genera_incertae_sedis* |  |
| -5,2597 | 0,0024 | *Shinella* |  |
| -3,0153 | 0,0429 | *Tepidimonas* |  |

**Table S3.** Significant differentially abundant phyla, classes, orders, families and genera between MT and MH samples. The table report the Log2FoldChange and adjusted p-values assessed using a paired Wilcoxon signed- rank test.
